# Editorial for Special Issue: “Thin Films Based on Nanocomposites (2nd Edition)”

**DOI:** 10.3390/nano14242049

**Published:** 2024-12-21

**Authors:** Marcela Socol, Nicoleta Preda

**Affiliations:** National Institute of Materials Physics, 405A Atomistilor Street, P.O. Box MG-7, 077125 Magurele, Romania

The continuous demand for multifunctional materials in industrial applications has driven the design of nanocomposites with new or enhanced properties. Hence, nanocomposite thin films have widespread use in technological applications including energy-related technologies, optoelectronic devices, and biomedical field. The present Special Issue includes nine research papers (eight articles and one review) focused on the development of various composites with properties in line with the targeted applications: functionalized natural fiber with enhanced mechanical performance [1], functionalized nanoparticles for drug delivery systems [2], coatings for osteogenesis [3], thin films for optoelectronic devices [4,5,6,7,8], and nitride films for tribology field [9].

Preda et al. developed hierarchical functionalized flax fibers with ZnO nanostructures via electroless deposition in order to enhance the interfacial adhesion between the natural fibers and synthetic matrix, the obtained ZnO nanostructured interphase allowing a smoother transition between the flax yarns and the epoxy resin matrix, which led to improvements in load transfer and mechanical interlocking [1].

Toderascu et al. prepared L-Cysteine (L-Cys)-coated magnetic iron oxide nanoparticles (NPs) loaded with doxorubicin (Dox) using a chemical method, the biological tests carried out on mouse and human metastatic melanoma cells evidencing the internalization of magnetic nanoparticles delivering Dox and highlighting the potential of these nanomaterials as efficient drug delivery vehicles in melanoma therapy [2]. 

Tokunaga et al. coated Ti implants (which are usually used in the osseointegration process) with ZrO_2_ using a molecular precursor method, changing the surface chemical composition of the metallic implants without changing the surface structure and roughness of the Ti implants, the deposited ZrO_2_ thin films promoting osteogenesis equivalent to or better than that of Ti in the early bone formation stage [3].

Rasoga and al. deposited mixed thin films based on arylenevinylene-based polymer donor and non-fullerene perylene diimide acceptor using Matrix Assisted Pulsed Laser Evaporation (MAPLE) on flat and nano-patterned aluminum (Al) electrodes; the results evidenced that the nanostructured metallic electrode induced an increase in the current value in the developed organic heterostructures, the achieved properties being suitable for optoelectronic devices [4].

Andreeva et al. used thin porous films of titanium dioxide (TiO_2_) impregnated with ions, molecular clusters, and silver (Ag) nanoparticles to fabricate highly ordered nanocomposite gratings using a commercially available nanosecond-pulsed UV laser system; the results emphasized their potential application for easier mass production of planar optical elements, polarization elements (splitters and filters), and hidden security labels [5]

Daryakar et al. analyzed the nonlinear optical response of metallic amorphous composite layers based on gold and iridium nanoparticles embedded in an equally nonlinear host matrix; the effective nonlinear susceptibility increased by orders of magnitude in the case of gold nanoparticles due to the localized surface plasmonic resonances [6]. 

Lu et al. investigated the nonlinear optical response of titanium nitride (TiN)/indium tin oxide (ITO) composites prepared via magnetron sputtering; the nonlinear absorption coefficient evaluated for the composite is about 14 times greater than that of single-layer TiN films due to the coupling of surface plasma between TiN and ITO [7].

Khan et al. fabricated Ni-doped aluminum nitride (AlN) thin films at room temperature using both direct current (DC) and radio frequency (RF) magnetron sputtering; the structural, morphological, photoluminescence, and optical properties recommended these nitride films for optoelectronic applications, especially in photovoltaic devices and lasers [8].

Wu et al. reviewed the strengthening and lubricating mechanisms in metal nitride films [9]. Their overview summarizes lubricating mechanisms including the easy-shear nature, tribo-chemical reactions, lubricious fluorides, the textured contact surface, and the synergistic effect [9]. Also, the influence of various parameters like the solid solution, the grain size, the secondary phase, the stress field, the template, and the valence electron concentration were analyzed for the design of nitride films with excellent hardness and lubricating performance [9].

As highlighted in this Special Issue, nanocomposites represent a ground-breaking class of materials that are able to provide solutions to industrial challenges ranging from electronics to the healthcare domain.
